# BCL-2 inhibitors in hematological malignancies: biomarkers that predict response and management strategies

**DOI:** 10.3389/fonc.2024.1501950

**Published:** 2025-01-21

**Authors:** Mariam Markouli, Maria N. Pagoni, Panagiotis Diamantopoulos

**Affiliations:** ^1^ Department of Internal Medicine, Boston Medical Center, Boston University School of Medicine, Boston, MA, United States; ^2^ Department of Hematology-Lymphomas and BMT Unit, Evangelismos Hospital, Athens, Greece; ^3^ First Department of Internal Medicine, Laikon General Hospital, National and Kapodistrian University of Athens, Athens, Greece

**Keywords:** Bcl-2 inhibitors, venetoclax, apoptosis, chronic lymphocytic leukemia, acute myeloid leukemia, DLBCL, BAX/BAK

## Abstract

Apoptosis is an essential characteristic of cancer and its dysregular promotes tumor growth, clonal evolution, and treatment resistance. B-cell lymphoma-2 (BCL-2) protein family members are key to the intrinsic, mitochondrial apoptotic pathway. The inhibition of the BCL-2 family pro-survival proteins, which are frequently overexpressed in B-cell malignancies and pose a fundamental carcinogenic mechanism has been proposed as a promising therapeutic option, with venetoclax (ABT-199) being the first FDA-approved BCL-2 inhibitor. Unfortunately, although BCL-2 inhibition has shown remarkable results in a range of B-cell lymphoid cancers as well as acute myeloid leukemia (AML), the development of resistance significantly reduces response rates in specific tumor subtypes. In this article, we explain the role of BCL-2 family proteins in apoptosis and their mechanism of action that justifies their inhibition as a potential treatment target in B-cell malignancies, including chronic lymphocytic leukemia, multiple myeloma, B-cell lymphomas, but also AML. We further analyze the tumor characteristics that result in the development of intrinsic or inherited resistance to BCL-2 inhibitors. Finally, we focus on the biomarkers that can be used to predict responses to treatment in the name of personalized medicine, with the goal of exploring alternative strategies to overcome resistance.

## 1 Introduction

Hematological malignancies are characterized by aberrant and persistent cellular proliferation in the bone marrow, lymph nodes, or blood. Clinical outcomes for blood cancers have significantly improved with the introduction of new targeted therapies such as, monoclonal antibodies, small molecule inhibitors, antibody-drug conjugates, bispecific T cell engagers, and, lastly, chimeric antigen receptor T (CAR-T) cells (1). However, relapsed or resistant disease is unavoidable with periods of remission getting increasingly shorter (2). For relapsed/refractory patients, novel treatment regimens targeting crucial pathways deleterious to tumor cells have started to emerge (3).

These include apoptotic pathways which are often dysregulated in cancer. In more details, malignant cells upregulate anti-apoptotic proteins in order to withstand the various genetic insults and pro-apoptotic changes. As a result, anti-apoptotic proteins, including those of the BCL-2 family (BCL-2, BCL-XL, BIM or Mcl-1), represent an attractive target for therapy (2). The BCL-2 protein is the direct target of Venetoclax (VTC) (ABT-199), a selective BCL-2 inhibitor that alters the mitochondrial apoptotic pathway and causes tumor cell death (4, 5). It has been proven effective in treating a range of hematological cancers (2). It is the first and only FDA-approved BCL-2 inhibitor to treat chronic lymphoytic leukemia (CLL) and some types of acute myeloid leukemia (AML). However, given the recent expansion in VTC applications, cases of VTC resistance have emerged, posing a major problem in clinical treatment.

In this article we describe the mechanisms of resistance to BCL-2 and focus on biomarkers that can be used to predict treatment responses. We also briefly discuss the treatment strategies that can be used to overcome this problem.

## 2 The BCL-2 regulated apoptotic pathway

The well-organized, genetically encoded mechanism known as apoptosis eliminates both damaged and no longer needed cells from the body (6). Unlike necrotic cell death, which is frequently uncontrolled and releases cellular debris that can cause tissue inflammation, apoptosis allows cells to be destroyed with little harm to neighboring cells (7). An essential characteristic of cancer is resistance to apoptosis, which promotes the growth of tumors, the formation of clonal cells, and treatment resistance (8, 9).

The BCL-2-regulated apoptotic route, which is sometimes referred to as the “intrinsic,” “stress,” or “mitochondrial” pathway, is tightly regulated by the interactions among the BCL-2 protein family members (10–14). The pro-survival BCL-2-like proteins (BCL-2, BCL-XL, BCL-W, MCL-1, A1/BFL-1), the multi-BH domain pro-apoptotic BAX/BAK proteins, and the pro-apoptotic BH3-only proteins (BIM, PUMA, BID, BAD, BIK, BMF, NOXA, HRK) make up this family of structurally related proteins (7).

**Figure 1:**
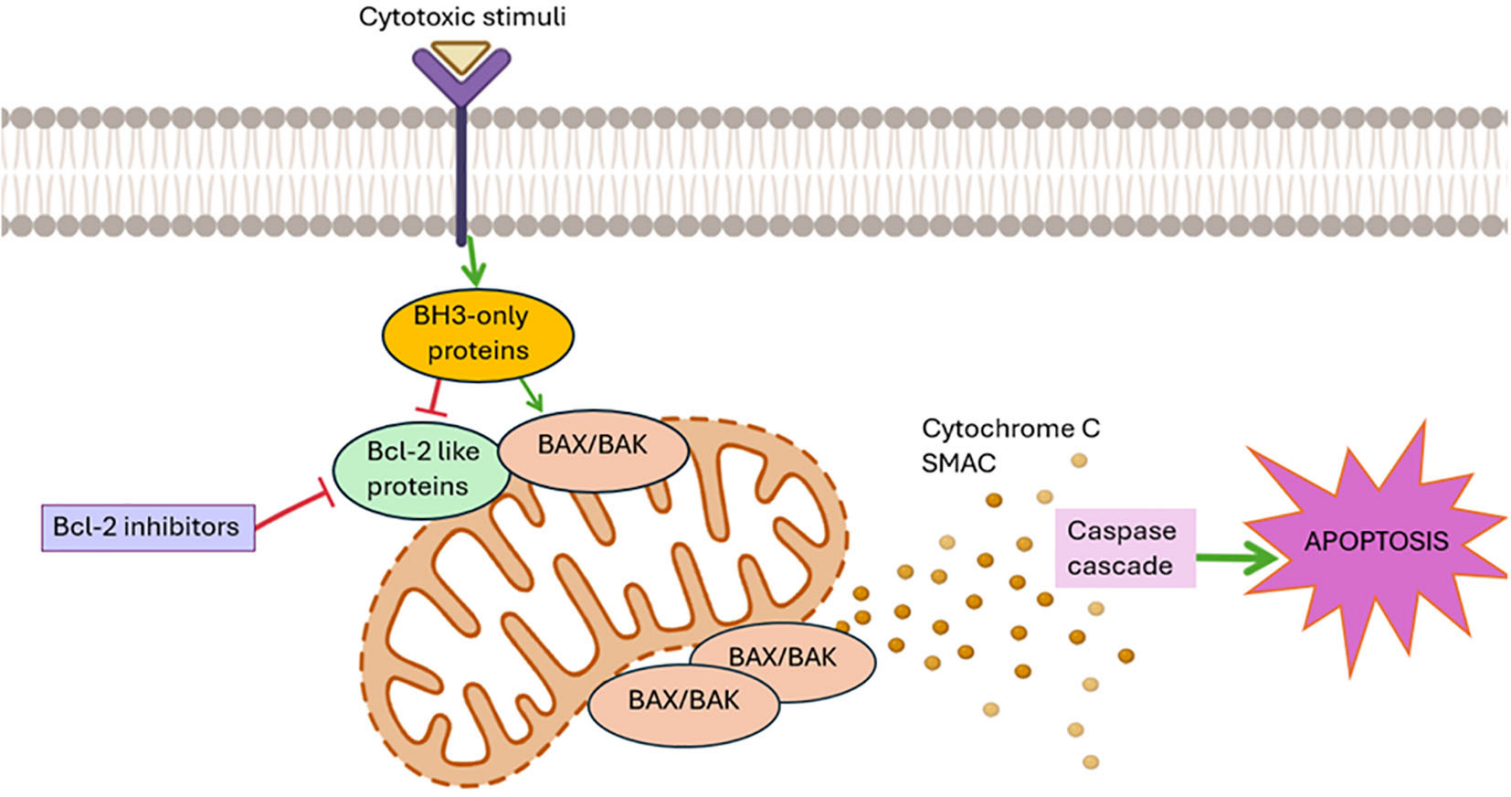
The Bcl-2-regulated apoptotic pathway: The pro-survival BCL-2-like proteins (BCL-2, BCL-XL, BCL-W, MCL-1, A1/BFL-1) inhibit the activation of the pro-apoptotic BAX/BAK proteins, thereby protecting the integrity of the mitochondrial outer membrane and promoting cell survival in healthy cells. In response to several cytotoxic stimuli, such as developmental signals, chemotherapeutic drugs, and genotoxic stress (15), the BH3-only pro-apoptotic proteins are activated and free BAX/BAK from the BCL-2-like proteins. When BAX/BAK are activated, they result in mitochondrial outer membrane permeabilization, which releases apoptogenic molecules such as cytochrome c and SMAC into the cytoplasm. This activates the caspase cascade, which ultimately leads to cellular destruction. In this context, numerous antagonists of anti-apoptotic proteins and particularly BCL-2 are being studied for the treatment of hematological neoplasms.

By inhibiting the activation of BAX and BAK, the BCL-2-like pro-survival proteins protect the integrity of the mitochondrial outer membrane and promote cell survival in healthy cells. In response to several cytotoxic stimuli, such as developmental signals, chemotherapeutic drugs, and genotoxic stress (15), the BH3-only proteins are either transcriptionally and/or post-transcriptionally activated to cause apoptosis by freeing BAX/BAK from the BCL-2-like protein’ inhibition or, in the case of some BH3-only proteins (such as BIM, tBID, and PUMA), by directly binding to BAX/BAK. When BAX/BAK are activated, they result in mitochondrial outer membrane permeabilization (MOMP), which releases apoptogenic molecules such as cytochrome c and SMAC/DIABLO into the cytoplasm. This activates the caspase cascade, which ultimately leads to cellular destruction (7) (**Figure 1**).

Cancer cells frequently overexpress antiapoptotic proteins, like BCL-2, BCL-XL, and MCL-1 which cause them to sequester large amounts of proapoptotic proteins in order to survive. When enough proapoptotic proteins are released, these cells are ready to undergo apoptosis; this condition is known as being “primed for death” (16). Numerous antagonists of anti-apoptotic proteins and particularly BCL-2 have therefore been produced and are being studied for the treatment of hematological neoplasms.

### 2.1 Therapeutic targeting of BCL-2

Apoptosis dysregulation in B-cell malignancies can be caused by overexpression of the BCL-2 protein. Follicular lymphoma (FL), CLL, one-third of diffuse large B-cell lymphoma (DLBCL), mantle cell lymphoma (MCL) and Waldenstrom macroglobulinemia (WM) exhibit aberrant BCL-2 expression. BCL-2 may trap some proapoptotic BCL-2 homology 3 (BH3)-only proteins (e.g., BIM, BID) to stop pore-forming proteins (BAX and BAK) from oligomerizing and leading to MOMP (mitochondrial outer membrane permeabilization) (15, 17). Since BCL-2 overexpression is a common finding in leukemias and lymphomas and because apoptosis blockage is a fundamental carcinogenic mechanism in lymphoid malignancies (7, 12)BCL-2 is considered a key therapeutic target.

Venetoclax (VCT/ABT-199) is the first highly selective oral BCL-2 antagonist licensed by the FDA for the treatment of relapsed/refractory chronic lymphocytic leukemia (CLL) with 17p deletion (18) and for patients age 75 and older with newly diagnosed AML or who have complications that made intensive induction chemotherapy ineligible. Venetoclax is a BH3 mimetic (9). It has also been used with azacytidine as a chemo-free bridge to transplantation (19).

In susceptible cells, it specifically targets the antiapoptotic BCL-2 protein and displaces the proapoptotic BIM from BCL-2 to activate effector proteins BAX or BAK, which then trigger apoptosis by the release of cytochrome c (9). The antiapoptotic MCL-1 and BCL-xL proteins provide resistance by sequestering BIM that has been shifted from BCL-2, and VTC does not target these proteins.

## 3 Development of resistance to BCL-2 inhibitors

While BCL-2 inhibitors have shown remarkable clinical results in a range of B-cell lymphoid malignancies, response rates to some subtypes of the disease are noticeably higher than those to others (e.g., CLL vs DLBCL) due to the level of initial BCL-2 dependence (intrinsic/innate or primary resistance) and/or development of resistance (acquired or extrinsic resistance) (15). In more detail, certain cell lines show higher BCL-2 expression and also BCL-2 expression-dependent sensitivity to BCL-2 inhibitors as explained below. Development of resistance on the other hand is regulated by several intracellular and microenvironmental variables, leading to heterogeneity in response rates (19).

### 3.1 Factors contributing to the development of primary resistance to BCL-2 inhibitors

#### 3.1.1 BCL-2 expression levels

One of the most important factors that may contribute to a tumor’s susceptibility to BCL-2 inhibition is the level of BCL-2 expression that is inherent to the tissue and its survival. Different tumor subtypes have different constitutional BCL-2 expression patterns, which can be caused by a number of different factors (20). The majority of CLL cells overexpress BCL-2 as a result of the *BCL-2 gene* hypomethylation, as well as loss of miR-15 and miR-16 at 13q14 and STAT3 transcription factor activation (21–23). Constitutive expression of BCL-2 (24) is caused by chromosomal translocation t(14;18)(q32;q21) in 80–90% of FL and 1/3 of DLBCL patients. Additionally, some somatic mutations raise BCL-2’s affinity for proapoptotic BH3-only proteins (25). In addition to BCL-2 translocation and amplification, double-hit lymphomas,which are a rare and aggressive subtype of DLBCL are characterized by *MYC* oncogene translocation with either the rearrangement of *BCL2* or *BCL6*. The enhanced expression of BCL-2 is also attributed to other processes, including phosphorylation, promoter hypermutation, and hypomethylation (26). As shown in the clinical experience treating FL, high levels of BCL-2 secondary to the translocation between chromosomes 14 and 18 were associated with poor responses to VTC monotherapy (15).

#### 3.1.2 Expression of other BCL-2 family proteins

However, upregulation of BCL-2 alone does not universally correlate with marked sensitivity to BCL inhibitor therapy (20). Therefore, deregulation of additional BCL-2 family proteins, such as BCL-W probably also play a role in the response to BCL-2 inhibiting therapies (20). Chromosome losses and somatic mutations impacting ATM, TP53, and NOTCH1 are examples of tumor heterogeneity that can impact therapeutic response rates and, primarily, the length of response (27, 28). Overexpression of BCL-2 family proteins is associated with certain baseline genomic abnormalities. It has been observed that CLL with trisomy 12 has decreased IRF4 transcriptional levels, increased NOTCH2 expression, and subsequently enhanced MCL1 expression (29). Similarly, abnormalities in the SWI-SNF chromatin remodeling complex are linked to the overexpression of BCL-XL in mantle cell lymphoma (30).

#### 3.1.3 Tumor microenvironment

In addition, intrinsic resistance in lymph nodes, the spleen, and other lymphoid tissue is probably a result of interactions between CLL cells and the surrounding microenvironment (31). Complementary microenvironmental niches can therefore serve to provide resistant subclones of heterogeneous CLL cells in lymphoid tissue a survival advantage (32). For example, microenvironmental stimulation, including T-cell engagement, probably causes an increase in baseline antiapoptotic BCL-2 family members such MCL1 (33, 34) and increased NF-κB signaling. Even when paired with anti-CD20, cells that have higher baseline levels of BCL-XL as a result of CD40 T-cell signaling are less susceptible to VTC (35).

### 3.2 Factors contributing to the development of acquired resistance to BCL-2 inhibitors

#### 3.2.1 Gene mutations and chromosomal abnormalities

**Figure 2:**
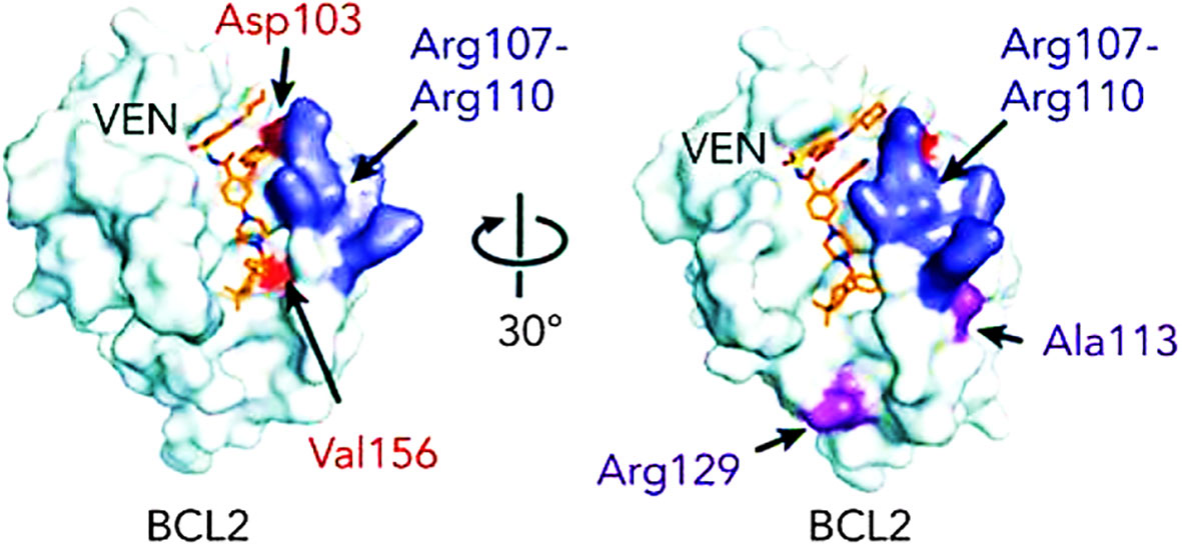
BCL-2 mutations that are linked to VTC resistance (38).

*BCL-2* mutations constitute one of the most common mechanisms of acquired resistance. One of the *BCL-2* genetic variants that is associated with resistance to VTC is Gly101Val, which predicts the failure of VTC to successfully remove the pro-apoptotic proteins from BCL-2 (36). When this mutation is acquired, CLL cell lines become resistant to the drug’s killing effects. This gives the mutant clone a selective advantage when it is continuously exposed to VTC (36). Other BCL-2 mutations that are linked to VTC resistance include alterations at the Asp103 codon resulting in amino acid substitutions to glutamic acid, valine, and tyrosine (37, 38). A number of other point mutations have been reported, including Arg107_Arg110dup, Leu119Val41, Arg129Leu40, and Val156Asp40, Phe104Leu and Phe104Ser, *BCL-2* Phe101Cys and Phe101Leu (38, 39). These mutations impact the sequence that separates the α2 and α3 helices. (**Figure 2**).

Along the Gly101Val variation, BCL-XL over-expression has been linked to a mutually exclusive mechanism of VTC resistance (38). A second clone exhibiting high BCL-XL, likely accounts for secondary VTC-resistance in the non-Gly101Val mutated cells.

After exhibiting VTC resistance, several CLL patient samples showed amplification of chromosome 1q [amp(1q23)], which causes dysregulations in energy metabolism and may be associated with VTC resistance. This region involves both *MCL1* and *PRKAB2*, which codes for an AMPK regulatory component, part of the AMPK pathway. These changes cause reprogramming of the mitochondria outer membrane biology and result in BCL-2 protein expression alterations (40).

#### 3.2.2 Post-transcriptional modifications

Further transcriptional and post-transcriptional modifications, such as phosphorylation have been identified as causing resistance to VTC by impacting the function of various BCL-2 family proteins. For example, in order for BAD, which inhibits BCL-2, BCL-XL, and BCL-w, to carry out its pro-apoptotic activities, it must be dephosphorylated at two distinct serine residues by simultaneous inhibition of mitogen activated protein kinase (MAPK) and phosphatidylinositol-3-kinase (PI3K) signaling (41). Another example is the stable monophosphorylation of MCL-1 after BCL-2 inhibition, whereas p53 activation induces dual-phosphorylation of MCL-1 by modulating MEK/ERK signaling, thereby targeting it to proteasomal degradation, overcoming resistance to VTC (42).

## 4 Biomarkers that can predict response to BCL-2 inhibitors

BCL-2 protein expression by itself is not enough to predict responses to VTC therapy; The development of genomic profiling methods and selective molecular targeted medicines has thankfully led to a significant growth in the utilization of biomarkers, in order to predict native/emergent resistance and treatment outcomes (35, 43) (**Table 1**).

**Table 1:** Biomarkers that can predict response to BCL-2 inhibitors.

Strategy	Biomarker	Reference
BCL-2 Mutations of the Venetoclax Binding Site	*-BCL-2*p.Gly101Val, -Phe104Cys/Phe104Leu -Ala113Gly -Arg129Leu -Arg107_Arg110dup -Asp103Tyr/Glu/Val -Val156Asp -Asp103Val(mutant D103Y)	(37, 44, 45)
Other BCL-2 family gene mutations	*-MCL1* gene amplifications on chromosome 1q21 -del(13q14)	(40, 46)
Detection of other gene mutations and cytogenic alterations	*-TP53* loss or mutations -loss of *CDKN2A/B*, *BTG1, SF3B1* and *NOTCH1* mutations -*IGHV* unmutated status -del(11q), del (13q), del (17p) - trisomy 12 with *NOTCH1*, *TP53*, and *FBXW7* gene alterations	(28, 47–49)
Observing the microenvironment	-detection of higher volumes of residual nodal disease after venetoclax treatment -detection of bulky lymphadenopathy prior to therapy	(50)

### 4.1 Measurement of BCL-2 family protein levels and modifications

The measurement of BCL-2 family protein levels can be utilized to predict responses to BCL-2 inhibitors. Expression of these proteins has been used in CLL lines *in vitro*, demonstrating that mostly BCL-XL and secondarily Mcl-1, and Bfl-1 overexpression causes complete resistance to single-agent treatment with VTC (35, 51). Moreover, measurement of elevated baseline MCL1 levels in CLL patients treated with VTC is related to worse progression-free survival (20). Elevated BFL-1 and BCL-W expression also appears to promote resistance to all combinations of BCL-2, BCL-XL and MCL-1 inhibitors, whereas BCL-2 levels alone can be measured to predict sensitivity to monotherapy with these drugs (40, 52, 53). Another study demonstrated that elevated MCL-1 and mainly BCL-2, BCL-2L1, and BCL-2L2 levels result in VTC resistance, thus having been proposed as useful biomarkers.

The development of kinase-mediated survival signals, including the phosphorylation of BCL-2, which causes a structural change in the VTC-binding groove has also been reported as an anti-apoptotic mechanism that is used by CLL cells to develop resistance (54).

### 4.2 Detection of BCL-2 mutations

#### 4.2.1 BCL-2 mutations of the VTC binding site

The detection of BCL-2 mutations has been shown to be critical in determining VTC resistance. These include *BCL-2* mutations that adversely alter the binding affinity to VTC (36, 55). The *BCL-2*p.Gly101Val mutation has been identified in patients that were initially responsive and then developed VTC resistance after receiving prolonged treatment from 19 to 42 months duration (36, 55). Of note, this mutation was not present before initiation of therapy. It affects a highly conserved residue that impacts the binding groove of VTC and reduces its affinity by 180-fold. Mutations in residue 104 also frequently appear in VTC-resistant leukemia and lymphoma lines where leucine or cysteine replaces phenylalanine (Phe104Cys/Phe104Leu), thereby impacting the BH3-binding groove (44). Other identifiable mutations include Ala113Gly, Arg129Leu, Arg107_Arg110dup, Asp103Tyr/Glu/Val, Val156Asp, all of which were not present before VTC therapy (37, 38, 45). On the other hand, the Asp103Val mutation (mutant D103Y) has been detected in patients refractory to VTC therapy (36).

#### 4.2.2 Other BCL-2 family gene mutations

As mentioned above, given VTC is very selective for BCL-2, one of the most important factors that may contribute to a tumor’s susceptibility to BCL-2 inhibition is the level of BCL2 expression that is inherent to the tissue and its survival. It’s possible that more BCL-2 family proteins, such as BCL-2, BCL-XL, BIM or Mcl-1 are overexpressed at rest and have higher affinity to pro-apoptotic BH3 proteins (20). Therefore, *MCL1* gene amplifications on chromosome 1q21.2 and chromosome 1q amplifications have been proposed to serve as biomarkers of response (40, 56, 57). Other mutations that influence BCL-2 regulators can also be exploited. For example, del(13q14) causing the loss of miR-15 and -16, which are negative posttranscriptional regulators of BCL-2 has been used in CLL to predict VTC responses (40).

#### 4.2.3 Detection of other gene mutations and cytogenic alterations

Mutations affecting non-BCL-2 family genes have also been proposed as potential biomarkers to identify shorter responses to VTC. These include *TP53* loss or mutations, loss of *CDKN2A/B*, *BTG1, SF3B1* and *NOTCH1* mutations, as well as *IGHV* unmutated status (58). There has been benefit in detecting these mutations early on during therapy in patients with relapsed/refractory CLL that develop progression (28, 58).

In a similar context, detection of chromosome 11q deletion, del(11q), that is linked to progressive CLL (47) helps predict that VTC monotherapy will not result in improvement or complete response (CR) (58). Similarly, trisomy 12 that allows for *NOTCH1*, *TP53*, and *FBXW7* gene alterations equals with minimal likelihood in achieving CR with VTC monotherapy in patients with relapsed or refractory CLL (47, 58). Chromosome 17p deletion, del(17p) is also worth mentioning in CLL patients given it has been considered the highest risk category, with the shortest OS and PFS (47). On the other hand, chromosome 13q deletion, del(13q) detection has been associated with an improved CR in patients receiving VTC monotherapy (58).

### 4.3 Observing the microenvironment

As mentioned above, microenvironmental stimulation, which includes T-cell engagement can have a great impact in the response to bcl-2 inhibitors. The clinical observation of nodal disease may thus help determine resistance. In more detail, patients with higher volumes of residual nodal disease after VTC therapy appear to have a shorter progression-free survival. Comparably, prior to therapy, bulky lymphadenopathy has been reliably linked to lower rates of full response and shorter response times to VTC (58).

## 5 Therapeutic strategies to overcome resistance

The abovementioned mechanisms of resistance can help guide the selection of therapeutic strategies that will help overcome resistance to BCL-2 inhibitors (**Table 2** **).**

**Table 2:** Medications that can be combined with BCL-2 inhibitors to overcome resistance.

Medication	Type of malignancy	Study phase	Reference
MCL-1 inhibition				
S63845			
AZD5991104	RR AML or MDS	I/II	NCT03466294
AMG176105			
MIK665	RR NHL, RR MM, RR AML	I	NCT04702425
Bortezomib	RR MM	III	NCT02755597
Dexamethasone	RR MM	III, II	NCT02755597, NCT02899052
Azacytidine	Elderly patients, previously untreated AML	II	NCT03466294
Cobimetinib	Elderly patients, RR AML	I	NCT02670044
Idelalisib			
Copanlisib	RR DLCBL	I/II	NCT04572763
Duvelisib	RR CLL or SLL or RS	I/II	NCT03534323
Dinaciclib	RR AML	I	NCT03484520
Epigenetic drugs				
Panobinostat			
NaB			
CUDC-907	RR B-cell lymphoma	I/II	NCT01742988
Bisantrene			
Targeting tumor cell metabolism				
Ritonavir			
Ionidamine			
2-DG			
Next generation BCL-2 inhibitors				
Sonrotoclax	CLL	III	NCT06073821
Navitoclax	RR CLL	I/II	NCT00481091
Obatoclax	RR NHL	I/II	NCT01238146
Tyrosine-kinase inhibitors				
Ibrutinib	CLL	II	NCT02756897

RR, relapsed/refractory; AML, acute myeloid leukemia; MDS, myelodysplastic syndrome; NHL, Non-Hodgkin’s lymphoma; MM, multiple myeloma; DLBCL, diffuse large B-cell lymphoma; CLL, chronic lymphocytic leukemia; SLL, small lymphocytic lymphoma; RS, Richter’s syndrome.

### 5.1 MCL-1 inhibition

Given one of the causes of innate resistance to VTC is the overexpression of MCL1, its inhibition may make cells more susceptible to BCL-2 inhibitors (59, 60). MCL1 targeting can be achieved by direct binding and inactivation or disruption of MCL-1 stability (59).

The first extremely potent and selective inhibitor found to bind to MCL-1’s BH3-binding groove was S63845. At effective concentrations, S63845 acts as a lone agent in multiple myeloma (MM) by preventing BAK and BAX from binding to MCL-1 while protecting normal tissues (61). By simultaneously inhibiting MCL-1 and BCL-2103, S63845 and VTC synergized *in vivo* in relapsed MCL and produced synthetic lethality. Furthermore, two other newly identified selective MCL-1 inhibitors, AZD5991104 and AMG176105, work in concert with VTC to cause MM cells to undergo apoptosis (62, 63). A phase Ib study is currently investigating the use of the BCL-2 inhibitor VOB560 and the MCL1 inhibitor MIK665 in hematological malignancies, to examine their safety and dosage (NCT04702425).

Furthermore, the proteasome inhibitor bortezomib has been combined with VTC, since it helps stabilize the MCL1-neutralizing protein NOXA and thereby indirectly inhibits MCL1 (64). Moreover, dexamethasone has the ability to upregulate BIM expression, which increases MM cells’ reliance on BCL-2 and, thus, their susceptibility to VTC (65). Phase I clinical trials have therefore tested the combination of VTC and dexamethasone, as well as VTC with bortezomib and dexamethasone with promising results (66, 67)., indicating that this three-drug combination regimen had good efficacy and mild adverse reactions. MCL1 expression can also be decreased by CDK9 inhibitors, such as flavopiridol and seliciclib (68, 69). When it comes to AML, VTC has been used in conjunction with azacitidine or decitabine, or low-dose cytarabine (70) with synergistic outcomes being attributed to the fact that azacytidine may cause MCL-1 downregulation (59, 71).

Finally, targeting pathways such as RAF/MEK/ERK (MAPK), PI3K/AKT/mTOR and CDK 2/Cyclin E that control MCL-1 expression on many different levels have recently been studied. It has been demonstrated that ERK1/2 increases the expression of the *MCL-1* gene and stabilizes protein expression (72). Therefore, MAPK-mediated MCL-1 expression inhibition has been proposed as a potential target to make cancerous cells more susceptible to VTC (59). Drugs that have been used in combination with VTC include cobimetinib (MEK inhibitor) Similarly, inhibiting the PI3K/AKT/mTOR pathway, which lowers MCL-1 levels (73), is an effective way to subtly overcome VTC resistance. In correlation with AKT-mediated BAX activation, cotreatment with the PI3Kδ inhibitor idelalisib and VTC boosted apoptosis and appears to decrease MCL-1 expression (74) VTC and the second-generation PI3K inhibitors copanlisib (75, 76) and duvelisib (77) may also work synergistically in malignant hematopoietic cells by downregulating MCL-1. Lastly, CDK2/Cyclin E-mediated MCL-1 phosphorylation inhibition by low dinaciclib concentrations sensitized ABT-737-resistant DLBCL cells to apoptosis by encouraging the release of sequestered BIM from MCL-1. Surprisingly, a strong synergistic activation of apoptosis has been shown when dinaciclib and VTC were combined in CLL and DLBCL cells (78). Alvociclib, a CDK9 inhibitor has also been studied in a phase Ib study together with VTC in patients with relapsed/refractory AML (NCT03441555).

### 5.2 Epigenetic drug combinations

Given the pervasive epigenetic dysregulations in hematological malignancies including DNA methylation, histone modifications, and chromatin remodeling, the use of epigenetic drug combinations also appears to be a promising approach. Thus, VTC, a pan-HDAC inhibitor called panobinostat and a MEK inhibitor have been proven to work together to efficiently cause apoptosis in MM cells. Mechanistically, MEK inhibition raises BIM levels, whereas panobinostat dissociates BIM/MCL-1 and BIM/BCL-xL complexes in MM cells by acting as a MCL-1 and BCL-xL inhibitor. Exposure to VTC then enhances BIM release from BCL-2, which in turn raises the expression of BIM/BAX and BIM/BAK complexes, to synergistically induce apoptosis (59). When it comes to AML, VTC’s effects are enhanced and resistance can be overcome when combined with sodium butyrate (NaB) administration, a short chain fatty acid derived from metabolism of dietary fibers. It appears that NaB may function as a HDAC inhibitor and is capable of upregulating BAX and BAK by cancelling the inhibitory effects of HDAC that is found on *Bax* and *Bak* genes. Similarly, fimepinostat (CUDC-907) is a dual inhibitor of HDAC and PI3K that can be used in combination with VTC in AML, whereas bisantrene is a topoisomerase-II inhibitor that can act as a HDAC inhibitor when combined with VTC and panobinostat (79, 80).

### 5.3 Targeting tumor cell metabolism

Malignant cells are dependent on glutamine and glucose, and different degrees of apoptosis are linked to the removal of either of those nutrients (81). The GLUT4 inhibitor ritonavir inhibits glucose metabolism, which prevents tumor cells from proliferating and causes death by lowering MCL-1 levels. This suggests that nutritional deprivation might restructure cellular reliance on BCL-2 family proteins and make them more susceptible to VTC (82). Furthermore, hexokinase inhibitors such lonidamine and 2-deoxyglucose (2-DG) increase ABT-263/737-induced apoptosis by indirectly lowering MCL-1 levels through the suppression of glycolysis and the depletion of ATP levels, which activates AMPK and inhibits MCL-1 (83, 84). Furthermore, it interferes with BAK’s interaction with MCL-1, which makes it easier for ABT-263/737 to free BAK from the MCL-1/BCL-xL/BAK121. Focusing on glutamine metabolism in relapsed/refractory MM patients sensitized MM cells to VTC by increasing expression of BIM and its binding to BCL-2 (85).

### 5.4 Next-generation BCL-2 inhibitors

As mentioned above, patients who have undergone VTC treatment and relapse may exhibit mutations that can impart resistance by interfering with chemical binding, such as G101V. Thus, there is a pressing need to create next-generation BCL2 inhibitors to overcome medication resistance. Sonrotoclax, a powerful and specific BCL2 inhibitor for example, is currently being studied and appears to exhibit more significant reduction of tumor development and higher cytotoxic action in different hematologic cancer cells compared to VTC (86). Navitoclax is another agent that demonstrates significant affinity for BCL-2 anti-apoptotic proteins, such as BCL-2, BCL-W, and BCL-XL (87). It has been tested in phase I and phase II clinical trials thus far and appears to effectively slow the growth of malignancies including acute lymphocytic leukemia (ALL) and the development of fibrosis in patients with myelofibrosis. It can either be used alone or in conjunction with other therapies where it can also improve the efficacy of other chemotherapeutic drugs (87). It was however associated with the dose limiting toxicity of thrombocytopenia that was BCL-X_L_-mediated This is why the BCL-X_L_–sparing and BCL-2–selective inhibitor, VTC, was developed (88). However recent preclinical studies are examining the administration of navitoclax as a galacto-conjugation prodrug (Nav-Gal), aiming to increase its tumor selectivity and minimize its toxic effects on platelets (89). Finally, obatoclax mesylate, a pan-BCL-2 protein family antagonist has been studied in phase I trials for leukemia and lymphoma. Although most patients showed hematological improvement with established well-tolerated regimens of obatoclax, fewer objective responses were documented. In addition, published phase II studies in myelofibrosis and lymphoma have not shown strong single-agent efficacy, suggesting that its potential can be achieved either through sensible combination treatments or when its use is directed by molecular predictors of response (90).

### 5.5 Tyrosine-kinase inhibitors

Malignant B cells proliferate excessively through B-cell receptor (BCR)–dependent signaling, which includes Bruton tyrosine-kinases. As mentioned above, kinase-mediated survival signals, including the phosphorylation of BCL-2, have also been reported as an anti-apoptotic mechanism that is used by CLL cells to develop resistance (54).

Detection of these changes may help guide treatment responses, including the addition of tyrosine kinase inhibitors (BTKi) to augment the cellular killing of VTC (54). In fact, the combination of BTKi with VCT has produced outstanding clinical results, with bone marrow undetectable minimal residual disease rates of around 66% in the frontline setting and 36% in relapsed CLL disease (91).

## 6 Conclusions- future directions

In this review, we discuss the use of BCL-2 inhibitors and their mechanism of action. It becomes evident that BCL-2 inhibitors can be used as a novel treatment option in hematological malignancies, however, their expanded use has led to both innate and acquired drug resistance. This must shift our therapeutic strategies to combination regimens, potentially as the initial treatment strategy to prevent the development of resistance. Biomarkers that predict treatment responses are therefore an absolute necessity and need to be further explored.

Future research and clinical trials are required and should continue to focus on identifying biomarkers that could help not only predict outcomes but also tailor therapeutic interventions and get around resistance mechanisms for each individual patient. By shedding more light on the complex interplay of BCL-2 family proteins, tumor cell metabolic reprogramming, or tumor microenvironment, we will be able to optimize BCL-2 inhibitor effectiveness by combining these medications with therapies that upregulate proapoptotic proteins and alter cellular metabolism.

Growing therapeutic options will need to be combined with sophisticated molecular testing, like BH3 profiling. This may allow the measurement of anti-apoptotic protein dependence and personalization of care before treatment is initiated, but also dynamic, individualized treatment adjustments for long-lasting illness remission.

*-Biorender was used to draft **Figure 1**,1.
